# Clinical significance and oncogenic function of NR1H4 in clear cell renal cell carcinoma

**DOI:** 10.1186/s12885-022-10087-4

**Published:** 2022-09-19

**Authors:** Shiyu Huang, Yanguang Hou, Min Hu, Juncheng Hu, Xiuheng Liu

**Affiliations:** 1 Department of Urology, Renmin Hospital of Wuhan University, Wuhan, 430060 Hubei China; 2 Institute of Urologic Disease, Renmin Hospital of Wuhan University, Wuhan, 430060 Hubei China; 3 Department of Cardiology, Renmin Hospital of Wuhan University, Wuhan, 430060 Hubei China

**Keywords:** NR1H4, ccRCC, Malignant phenotype, CCNE2, Diagnosis, Prognosis, Multi-omics, Immunotherapy

## Abstract

**Background:**

Nuclear receptor subfamily 1 group H member 4 (NR1H4) have been reported in various cancer types, however, little is known about the clinical values and biological function in clear cell Renal cell carcinoma (ccRCC).

**Methods:**

The expression pattens of NR1H4 in ccRCC were investigated in clinical specimens, cell lines and publicly‑available databases. Cell Counting Kit-8 (CCK-8), colony formation, 5-ethynyl-2' -deoxyuridine (EdU), transwell and cell wound healing assays were performed to assess the biological functions of NR1H4 in 786-O ccRCC cells. Gene set enrichment analysis (GSEA), Flow Cytometry, quantitative real‐time PCR (qRT-PCR), western blot and immunofluorescence were performed to explore the molecular mechanism of NR1H4 in ccRCC. We explored the early diagnostic value, prognostic value, genetic mutation and DNA methylation of NR1H4 by a comprehensive bioinformatics analysis based on the data published in the following databases: The Cancer Genome Atlas (TCGA), Gene Expression Omnibus (GEO), Kaplan‐Meier Plotter, Gene Expression Profiling Interactive Analysis (GEPIA), UNIVERSITY OF CALIFORNIA SANTA CRUZ Xena (UCSC Xena), cBio Cancer Genomics Portal, MethSurv, SurvivalMeth and The University of ALabama at Birmingham CANcer data analysis Portal (UALCAN). Its correlation with tumor-infiltrating immune cells in ccRCC was analyzed by Tumor Immune Estimation Resource 2.0 (TIMER2.0) and Tumor Immune System Interactions Database (TISIDB).

**Results:**

In this study, NR1H4 was found to be highly expressed in ccRCC tissues and ccRCC cell lines. Knockdown of NR1H4 significantly suppressed cancer cell proliferation, migration and invasion. Mechanistically, tumor‐associated signaling pathways were enriched in the NR1H4 overexpression group and si-NR1H4 could induce the downregulation of Cyclin E2 (CCNE2). By bioinformatics analysis, NR1H4 was identified as highly expressed in stage I ccRCC with a high diagnostic accuracy (area under the receiver operating characteristic curve > 0.8). Genetic alteration and DNA methylation of NR1H4 were significantly associated with prognosis in ccRCC patients. Moreover, NR1H4 expression associated with immune cell infiltration levels in ccRCC, which provides a new idea for immunotherapy.

**Conclusions:**

Our study indicated that NR1H4 might be a potential tumor biomarker and therapeutic target for ccRCC which could promote cancer cell proliferation, migration and invasion via regulating CCNE2.

**Supplementary Information:**

The online version contains supplementary material available at 10.1186/s12885-022-10087-4.

## Background

ccRCC, or kidney renal clear cell carcinoma (KIRC), is the most common subtype of renal cell carcinoma, with a prevalence of 70% among all renal cell carcinoma [1]. Global cancer statistics of 2020 indicates that renal cell cancer is the 16th most common cancer in the world, which accounting for 2.2% of the total cancer incidence with 431,288 new cases and 1.8% of the total case mortality with 179,368 deaths annually [2]. ccRCC usually accompanied by high metastasis rate and high mortality, but patients diagnosed and treated in an early stage showed better outcomes. With current diagnostic methods, such as Computed Tomography and Magnetic Resonance Imaging, there are still approximately 15% RCC patients have progressed into distant metastasis at clinical diagnosis, resulting in poor prognosis [3, 4]. Therefore, identifying effective early diagnosis and prognosis biomarkers and therapeutic target of ccRCC is urgently needed.

NR1H4, also called farnesoid X receptor (FXR) is a transcription factor belonging to the superfamily of nuclear receptors [5, 6]. NR1H4 involved in several biological processes, including lipogenesis, gluconeogenesis, ammonia detoxification, glycogen synthesis, bile acid metabolism and inflammation [7, 8]. Recently, NR1H4 has been reported in multiple cancers, including colorectal cancer (CRC), hepatocellular carcinoma (HCC), non-small cell lung cancer (NSCLC), breast cancer, cholangiocarcinoma, cervical cancer [9–14]. Shan Li. et al. reported that activation of NR1H4 induces antitumor activity in colorectal cancer by suppressing JAK2/STAT3 signaling via transactivation of Suppressor of cytokine signaling 3 (SOCS3) gene [15]. Mi Chen. et al. reported that NR1H4 directs asymmetric cell division of Sox9 + cells to prevent the development of liver cancer via Notch1 in a mouse model [16]. However, the function of NR1H4 in ccRCC has not yet been completely elucidated.

Abnormal expression of cyclins affects the cell cycle and eventually leads to the progression of cancer. CCNE2 and its associated catalytic partners, cyclin-dependent kinase 2 (CDK2), have important functions in cellular biological processes [17, 18]. CCNE2 overexpression is observed in a variety of cancers, including non-small cell lung cancer (NSCLC), bladder cancer, leukemia and breast cancer [17, 19–21]. Nevertheless, the correlation between CCNE2 and NR1H4 in the development and progression of ccRCC remains elusive.

In the present study, we reported that NR1H4 was overexpressed in ccRCC and high expression of NR1H4 is important to promote tumor progression. Moreover, we first assessed its correlation with clinical prognosis, early diagnosis and infiltrating immune cell in ccRCC through bioinformatics analysis. Our data revealed a novel regulator in ccRCC development and implicated the clinical significance of NR1H4.

## Materials and methods

### Publicly‑available databases analysis

RNA-seq data and clinical information for ccRCC were obtained from TCGA (https://portal.gdc.cancer.gov), including 538 tumor samples and 72 adjacent normal tissues' transcriptome data and clinical information of the corresponding patients.

Four sets of microarrays (GSE46699, GSE167093, GSE40435 and GSE126964) were downloaded from the GEO database (https://www.ncbi.nlm.nih.gov/geo) and used for the validation. Table 1 lists the details of datasets.

**Table 1:** The sample information of five datasets in Gene Expression Omnibus Database

GEO Accession No	Platform	Normal sample	Tumor sample	Total sample
GSE46699	GPL570	63	67	130
GSE167093	GPL10558	254	604	858
GSE40435	GPL10558	101	101	202
GSE126964	GPL20795	11	55	66

TIMER2.0 (http://timer.cistrome.org/), a web server for comprehensive analysis of tumor-infiltrating immune cells [22], was used to investigate correlations between NR1H4 expression and tumor-infiltrating immune cells and the expression of the NR1H4 across all TCGA tumors.

GEPIA (http://gepia.cancer-pku.cn/index.html), an analysis tool containing RNA sequence expression data of 9,736 tumors and 8,587 normal tissue samples [23], was used to assess the expression pattern and prognostic value of NR1H4 in ccRCC.

UALCAN (http://ualcan.path.uab.edu/index.html), an interactive web resource [24], was used to further investigate the clinicopathological insights and promoter methylation of NR1H4.

The Kaplan‐Meier Plotter (http://www.kmplot.com) and UCSC Xena (https://xena.ucsc.edu/) were used to assess the prognostic value of NR1H4 in ccRCC [25, 26].

The cBio Cancer Genomics Portal (http://cbioportal.org), a comprehensive web resource for interactive exploration of multidimensional cancer genomic datasets [27, 28], was used to analyze NR1H4 alterations in the TCGA KIRC sample.

MethSurv (https://biit.cs.ut.ee/methsurv/) was used to assess the DNA methylation level of NR1H4 and SurvivalMeth (http://bio-bigdata.hrbmu.edu.cn/survivalmeth/) was used to assess the prognostic value of the DNA methylation of NR1H4 in patients with ccRCC [29, 30].

#### TISIDB (http://cis.hku.hk/TISIDB/index.php), a web portal for tumor and immune system interactions, was used to validate correlations between NR1H4 expression and tumor-infiltrating immune cells.

### Clinical samples, cell culture and treatment

ccRCC tissues and adjacent normal tissues were obtained from patients undergoing urological surgery in Wuhan University People’s Hospital. The privacy rights of human subjects always be observed. Human ccRCC cell lines 786-O, 769P, A498, ACHN and normal kidney tubular epithelial cell HK-2 were obtained from the ATCC (American Type Culture Collection). HK-2 was cultivated in DMEM medium (Cytiva, Logan Utah, USA) supplemented with 10% fetal bovine serum (FBS) (GIBCO, Grand Island, NY, USA) and ccRCC cells were cultured in RPMI 1640 medium (Cytiva, Logan Utah, USA) supplemented with 10% FBS. All cells were cultured in the same humidified atmosphere (37 °C with 5% CO2). 786-O cell line was transfected by NR1H4-specific siRNA for 6 h. Meanwhile, nontargeting siRNA (Sangon Biotech, Shanghai, China) was used to transfect 786-O cells as a negative control. The siRNA transfections were performed using Lipo6000 transfection reagent (Beyotime, Shanghai, China), according to the manufacturer’s protocol. After 48 h, cell experiments were performed.

### Quantitative real‐time PCR and western blot

Total RNA was isolated using the TRIzol reagent (Invitrogen, Carlsad, CA, USA) and was reversely transcribed to cDNA with the PrimeScript™ RT Reagent Kit with gDNA Eraser (TaKaRa, Shiga, Japan). qRT-PCR was performed on the Roche LightCycler 480 detection system with TB Green Premix Ex Taq II (TaKaRa, Shiga, Japan) and gene expressions were normalized to GAPDH. The quantitative analysis was calculated by using 2 − ΔΔCt method. The primers were all listed in Additional file 1: Table S1.

For western blot, proteins were separated on 10% SDS-PAGE gels (50 mg/lane) and then transferred to PVDF membranes (Sigma-Aldrich, St. Louis, MO, USA). The membranes were blocked with 5% non-fat milk in TBST buffer (10 mmol/L Tris–HCl, i0.15 mol/L NaCl, and 0.05% Tween20, pH 7.2) for 1 h and incubated with primary antibodies overnight at 4 °C. Primary antibodies used here were monoclonal mouse antibody against NR1H4/FXR (1:1,000 dilution; Santa Cruz Biotechnology, Dallas, Texas, USA, sc-25309), monoclonal mouse antibody against Cyclin E2 (1:1,000 dilution; Santa Cruz Biotechnology, Dallas, Texas, USA, sc-28351), polyclonal rabbit antibody against CDK2 (1:1,000 dilution; Wanleibio, Shenyang, China, WL01543) and polyclonal rabbit antibody against GADPH (1:1,000 dilution; Servicebio, Wuhan, China, GB11002). After extensive washing with TBST buffer, the membranes were incubated with anti-mouse (1:5,000 dilution; Servicebio, Wuhan, China, GB23301) or anti-rabbit (1:5,000 dilution; Servicebio, Wuhan, China, GB23303) IgG secondary antibody at room temperature for an additional 1 h. Protein bands were scanned by the ChemiDoc™ XRS + system and analyzed using an Image Lab software (Bio-Rad Laboratories, Inc.).

### Flow cytometry

Cell cycle analysis was performed by flow cytometry with PI staining. 786-O cells (1 × 10^6^ cells/ml) with different pretreatments were seeded into 6‐well plate and fixed with 75% ethanol overnight. Cells were then resuspended with cold PBS and stained with 100 μg/ml of RNase A and 50 μg/ml of PI for 30 min in the dark. CytoFLEX (Beckman Coulter Biotechnology, Suzhou, China) was used to analyze the DNA content of cells.

### Immunohistochemistry and immunofluorescence

For immunohistochemistry (IHC), the paraffin-embedded tissues were cut into 5 μm sections and were incubated with specific antibodies for NR1H4 (mouse monoclonal, 1:50, Santa Cruz Biotechnology, Dallas, Texas, USA, sc-25309) overnight at 4 °C, then incubated with secondary antibody for 30 min at 37 °C, followed by the addition of a stain. For immunofluorescence, the cells of different treatments were fixed with 4% paraformaldehyde and permeabilized with 0.1% Triton X-100/PBS. After washing with PBS, they were blocked with 10% goat serum for 1 h at room temperature and incubated with mouse monoclonal antibody against Cyclin E2 (1:1,000 dilution; Santa Cruz Biotechnology, Dallas, Texas, USA, sc-28351) for 2 h. After several washes with PBS, the cells were treated with the secondary antibody (goat anti-rabbit) for 1 h at 37 °C. The nuclei were stained with DAPI. Images were collected under microscope.

### Cell proliferation assay

For CCK8 assay, cells were seeded in a 96‐well plate at the density of 2,000 cells per well. The cell viability was detected at four selected time points (0, 12, 24 and 48 h). CCK‐8 solution (10μL) was added to each well at indicated times and incubated for another 3 h. The optical density values were detected at 450 nm (Bio-Rad Laboratories, California, Hercules, USA).

For colony formation assay, cells were seeded into 10 cm plate with 1000 cells/well. When there were at least 50 cells under microscope for single clone after around 2 weeks, clones were fixed using 4% paraformaldehyde and stained using 0.1% crystal violet. After washed and aired, clones were pictured.

For EdU assay, the EdU kit (Click-iT EdU-594 Cell Proliferation Kit, Servicebio, Wuhan, China) was used. 786-O cells (2 × 10^4^ cells/well) were seeded in 24-well plates. Subsequently, cells were incubated with EdU for 2 h, fixed with 4% paraformaldehyde and stained the nuclear with Hoechst. Images were collected under microscope.

### Cell migration and invasion assay

For transwell assay, 1 × 10^5^ cells in medium without FBS were seeded onto the upper 24-well transwell chamber containing an uncoated or Matrigel-coated membrane. Next, 600 µl medium containing 30% FBS was placed into the lower chambers. After 24 h, the cells that crossed the inserts were fixed with 4% paraformaldehyde and stained with 0.1% crystal-violet. Three fields in each well were randomly chosen to count migrated and invaded cells via microscope (200 × magnification).

For wound healing assay, cells were seeded into 6‐well plates. After the cells reached 80%‐90% confluence, the cell layer was scratched with a 10 µl pipette tip and the medium containing 10% FBS was replaced with a serum-free medium. The wounds were observed and imaged at 0 and 24 h after wounding.

### Gene set enrichment analysis

GSE167093 with a functional gene set file (c2.cp.kegg.v7.4.symbols.gmt) was analyzed by GSEA to obtain pathways enriched by NR1H4. High (top 50%) and low (bottom 50%) NR1H4 expression groups were divided according to the median expression of NR1H4. Gene sets with nominal p-value less than 0.05 and FDR less than 0.25 were considered of statistically significant.

### Statistical analysis

All data were processed using the R software (version3.6.0), GraphPad 8.0, and SPSS version 23.0 (SPSS). Paired t‑test, unpaired t-test and one‐way ANOVA followed by Tukey post hoc test were used to compare the expression of NR1H4 in different groups. A Chi-square test was performed to analyze the correlation of NR1H4 expression and clinicopathological factors. The diagnostic value of NR1H4 in lung cancer was revealed by receiver operating characteristic (ROC) curves. Univariate and multivariate survival analyses were executed using the Cox proportional hazards regression model. All experiments were performed at least three times; data are presented as mean ± SD. P values less than 0.05 were considered statistically significant.

## Results

### NR1H4 is overexpressed in ccRCC and correlated with clinicopathological features

**Fig. 1:**
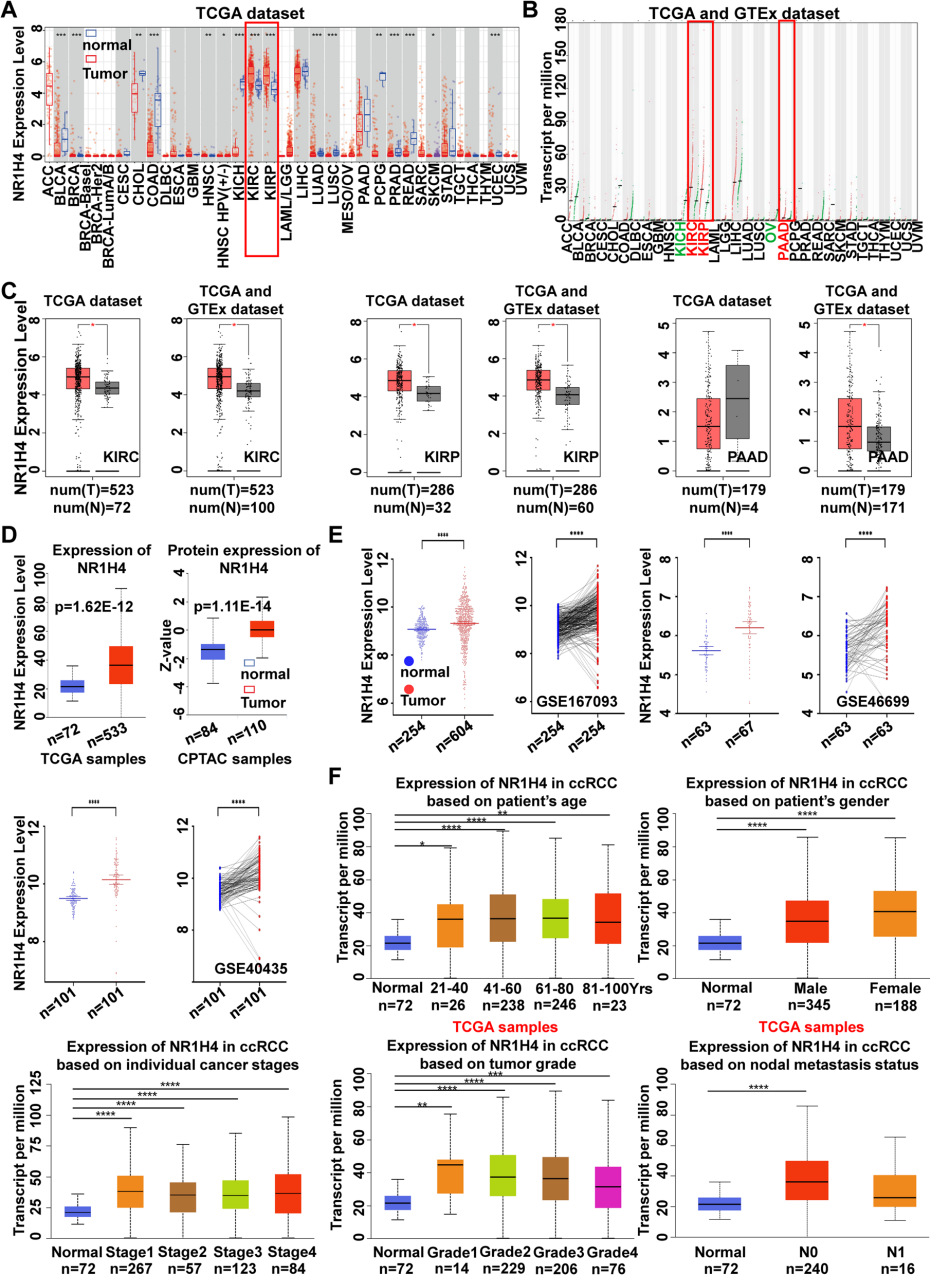
NR1H4 was overexpressed in ccRCC. **A** NR1H4 expression levels in different tumor types from TCGA dataset were determined by TIMER2.0 database. **B** NR1H4 expression levels in different tumor types from TCGA and GTEx dataset were determined by GEPIA database. **C** NR1H4 mRNA expression level in KIRC, KIRP and PAAD. **D** NR1H4 mRNA and protein expression in ccRCC by the UALCAN database. **E** Expression of NR1H4 in ccRCC and normal tissues in GEO database, including GSE167093 (*n* = 858), GSE46699 (*n* = 130) and GSE40435 (*n* = 202). **F** Expression analysis of NR1H4 based on different variables including patient age, patient gender, individual cancer stages, tumor grade and nodal metastasis status. NR1H4, Nuclear receptor subfamily 1 group H member 4; ccRCC, clear cell renal cell carcinoma; TIMER, Tumor Immune Estimation Resource; KIRC, kidney renal clear cell carcinoma; KIRP, kidney renal papillary cell carcinoma; PAAD, pancreatic adenocarcinoma; GEO, Gene Expression Omnibus; TCGA, The Cancer Genome Atlas; GAPDH, glyceraldehyde-3-phosphate dehydrogenase; *p < 0.05; **p < 0.01; ***p < 0.001; ****p < 0.0001

**Fig. 2:**
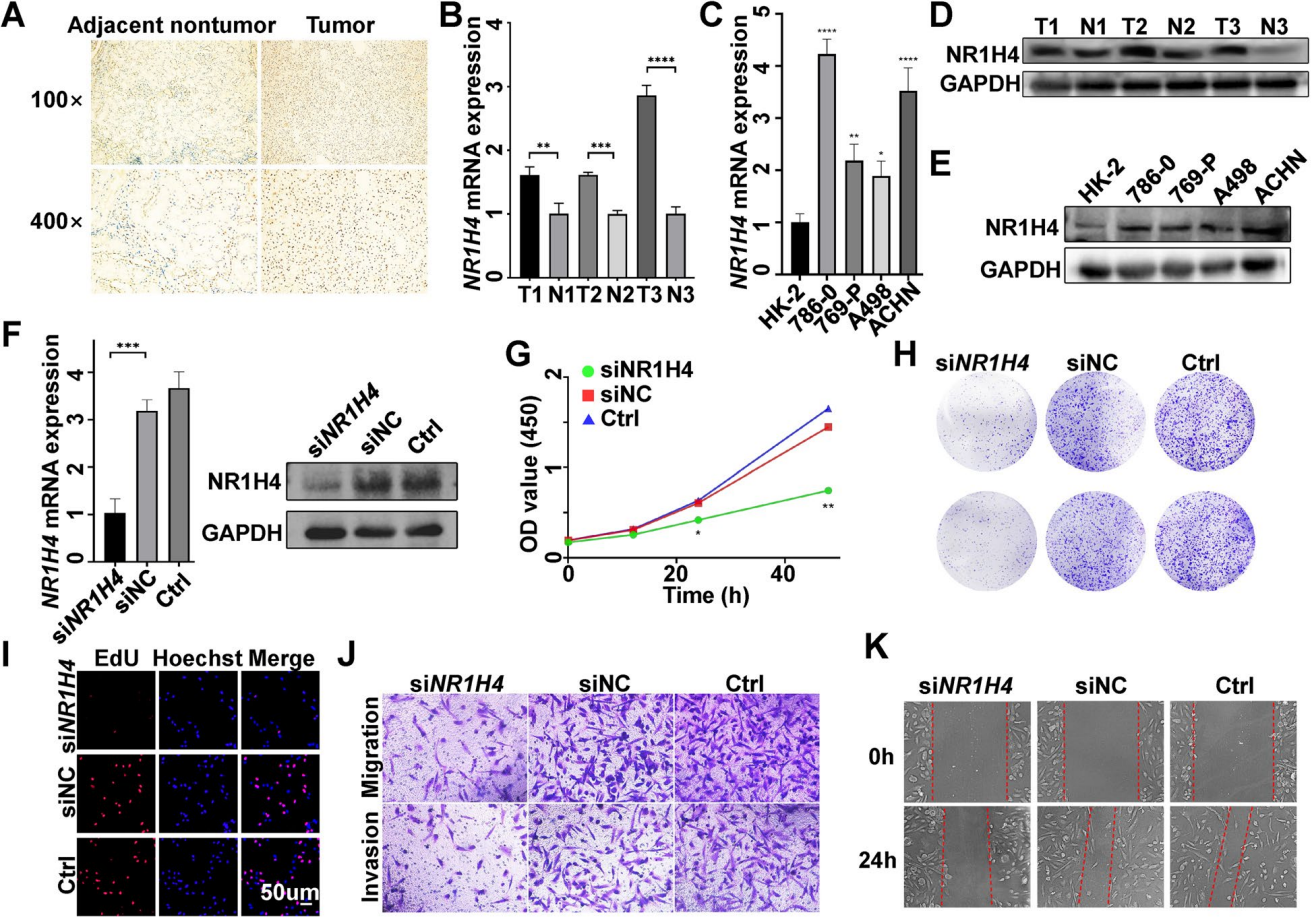
Knockdown of NR1H4 suppressed proliferation, migration and invasion of ccRCC cells. **A** Immunohistochemistry assay showed the higher expression of NR1H4 in ccRCC tumor tissues when compared with tumor adjacent tissues. **B-E** Analysis of NR1H4 expression in 3 paired ccRCC specimens and ccRCC cell lines through quantitative real‐time PCR and western blot. **F** qRT-PCR and western blot verified the knockdown of NR1H4 in 786-O cells via siRNA. NR1H4 downregulation inhibited proliferation of 786-O cells, as demonstrated by (**G**) CCK-8 assay, (**H**) clone formation assay and (**I**) EdU assay. NR1H4 knockdown attenuated the migration and invasion ability of ccRCC cells indicated by transwell assay (**J**) and wound healing assay (**K**). OD, optical density; EdU (5-ethynyl-2' -deoxyuridine); *p < 0.05; **p < 0.01; ***p < 0.001

Abnormal expression of NR1H4 has been reported in various cancers[14, 31]. However, studies on the functional role of NR1H4 in ccRCC are lacking. Data from TIMER2.0 and GEPIA revealed that the overexpression of NR1H4 was detected in KIRC and kidney renal papillary cell carcinoma (KIRP) which indicated NR1H4 was a novel oncogene (Fig. 1A-C). Due to KIRC is the most common subtype of renal cell carcinoma, we analyze the expression pattern of NR1H4 in CPTAC samples and GEO samples with ccRCC, which was identical with the result before (Fig. 1D, E). In addition, TCGA samples exhibited that NR1H4 was upregulated in all the variables compared to the normal, including patient’s age, patient’s gender, individual cancer stages, tumor grade, nodal metastasis status (Fig. 1F). GSE167093 was used to investigate the correlation between NR1H4 expression and clinicopathological features of ccRCC, we found higher expression levels of NR1H4 significantly contributed to tumor stage (Table 2). Furthermore, the expression of NR1H4 was detected in clinical specimens both on mRNA and protein level. The results of IHC revealed the higher expression of NR1H4 in ccRCC samples in contrast to tumor adjacent samples (Fig. 2A). Similarly, Fig. 2B and Fig. 2D showed that NR1H4 was overexpressed in ccRCC tissues both on mRNA and protein level compared to adjacent nontumor tissues. The expression of NR1H4 was further investigated in ccRCC cell lines using qRT-PCR and western blot, which corroborated the results above (Fig. 2C,E).

**Table 2:** Correlation between NR1H4 expression and the clinicopathological features of ccRCC patients in GSE167093

Characteristics	No. of patients	NR1H4 expression		Chi square value	*p*-value
		Low	High		
Age (years)				2.713	0.119
≤ 55	170	81	89		
> 55	432	174	258		
Gender				1.499	0.241
Male	357	144	213		
Female	247	112	135		
Stage				10.785	0.013
1	306	123	183		
2	98	55	43		
3	138	58	80		
4	62	20	42		
Grade				5.013	0.171
1	100	47	53		
2	304	115	189		
3	105	36	69		
4	24	12	12

### Knockdown of NR1H4 suppressed proliferation, migration and invasion of ccRCC cells

According to previous reports, inhibition of NR1H4 has an anti-cancer effect [32]. To explore the effect of NR1H4 on the function of ccRCC cells, we used siRNA to suppressed NR1H4 expression in 786-O cells (Fig. 2F). CCK-8, clone formation, and EdU assays were used to investigate the influence of NR1H4 on ccRCC cell proliferation. The results of CCK-8 assay indicated that the proliferation of si-NR1H4 transfected cancer cells was significantly reduced compared to negative control transfected cells (Fig. 2G). The number of cell clones and the percentage of EdU positive stained cells in NR1H4 downregulated cells was less than that in control cells (Fig. 2H, I). Suppression of NR1H4 also caused decline of the 786-O cells migration and invasion ability as indicated by transwell assays and wound healing assay (Fig. 2J, K). The results suggest that NR1H4 could promote ccRCC cell proliferation, migration and invasion in vitro.

### NR1H4 regulates the expression of CCNE2, CDK2

**Fig. 3:**
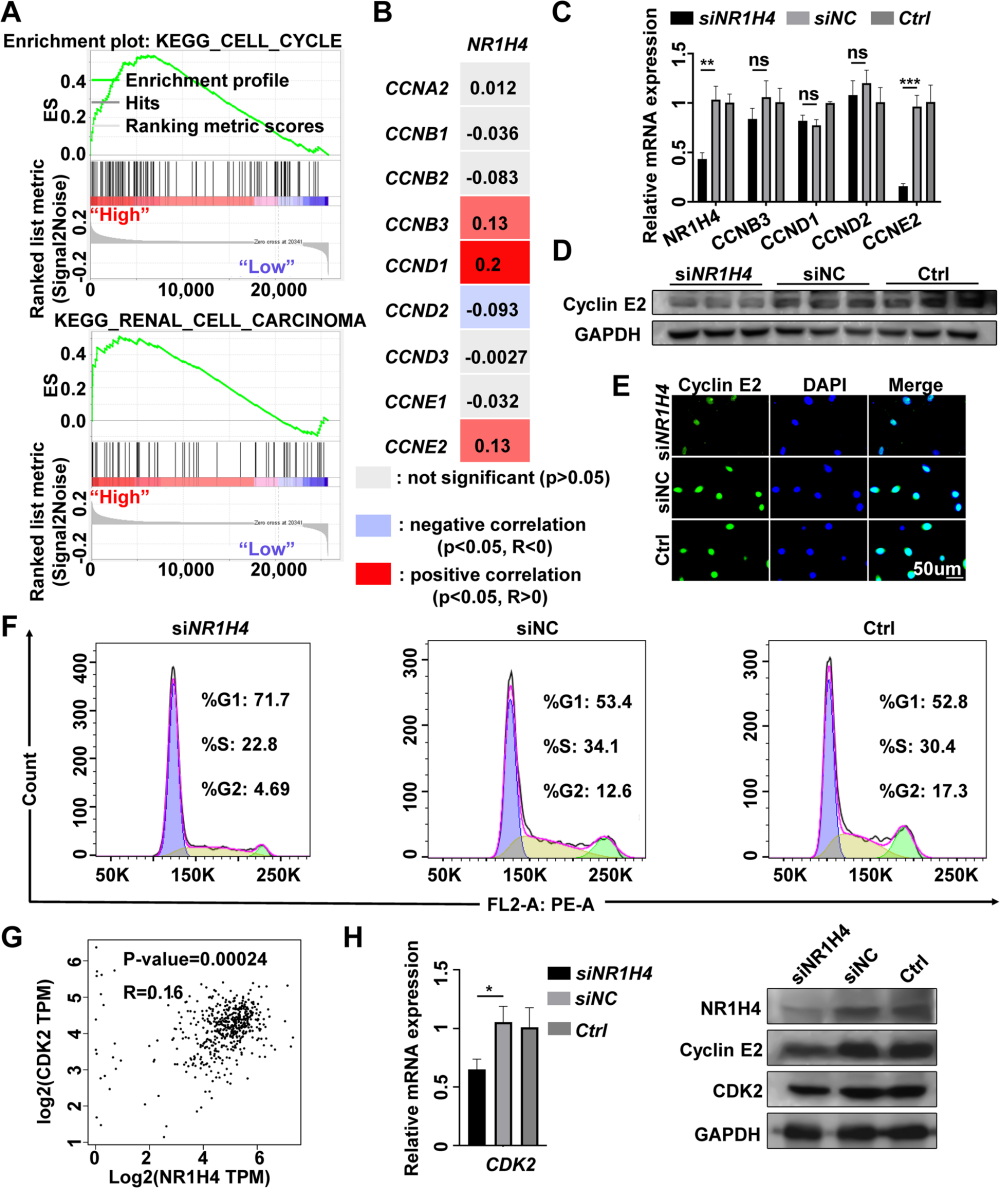
NR1H4 regulates the expression of CCNE2, CDK2. **A** Cell cycle was enriched in the NR1H4 high expression group of ccRCC. **B** The correlation analysis for NR1H4 and the Cyclins family members in TCGA database. **C** The mRNA levels of CCNE2 was downregulated in NR1H4 knockdown cells by qRT-PCR assays. The protein levels of CCNE2 was downregulated in NR1H4 knockdown cells by western blot (**D**) and immunofluorescence (**E**). **F** The distribution of cells in different cell cycle stages was analyzed by Flow Cytometry. **G** Scatterplots of correlations between NR1H4 expression and CDK2 which derived from an analysis of the TCGA dataset. (**H, I**) qRT-PCR and western blot revealed that knockdown of NR1H4 inhibited CDK2 mRNA and protein level in 786-O cells. CCNA2, cyclin A2; CCNB1, cyclin B1; CCNB2, cyclin B2; CCNB3, cyclin B3; CCND1, cyclin D1; CCND2, cyclin D2; CCND3, cyclin D3; CCNE1, cyclin E1; CCNE2, cyclin E2; CDK2, cyclin dependent kinase 2; DAPI, 2-(4-Amidinophenyl)-6-indolecarbamidine dihydrochloride; *p < 0.05; **p < 0.01; ***p < 0.001

GSE167093 has the largest sample size, so we performed GSEA on the GSE167093 dataset by comparing the high and low NR1H4 expression groups to further verify the associated signaling pathways activated in ccRCC. Table 3 listed the top 20 enrichment results. High (top 50%) and low (bottom 50%) NR1H4 expression groups were divided according to the median expression of NR1H4. The results showed that cell cycle, mismatch repair, DNA replication, nucleotide excision repair, renal cell carcinoma, mTOR signaling pathway were enriched in the NR1H4 highly expressed group, which provides a potential mechanism whereby NR1H4 might regulate the progression of ccRCC. Cell cycle signaling pathway was enriched in the NR1H4 highly expressed group with the biggest size (Fig. 3A). Cyclins regulate a wide range of cellular functions, inhibiting cell-cycle proteins may contribute to cancer therapy [33]. The TCGA dataset was analyzed to further evaluate the correlations of NR1H4 expression with cyclins, Fig. 3B showed that NR1H4 expression was associated with CCNB3 (R = 0.13, *p* = 0.0041), CCND1 (R = 0.2, *p* = 4.1e-6), CCND2 (R = -0.093, *p* = 0.033) and CCNE2 (R = 0.13, *p* = 0.002) expression. The results of qRT-PCR indicated that si-NR1H4 could induce the downregulation of CCNE2 rather than other cyclins (Fig. 3C). Western blot and immunofluorescence analysis further showed that NR1H4 knockdown was associated with downregulation of Cyclin E2 (Fig. 3D, E). The main function of Cyclin E2 is to help cells switch from G0/G1 to S phase by binding of CDK2 (the catalytic partners of Cyclin E2). By Flow Cytometry, NR1H4 knockdown resulted in less cell cycle progression, with an accumulation of cells in the G0/G1 phase and a decrease of cells in the S-phase (Fig. 3F). Moreover, NR1H4 expression was positively correlated with CDK2 expression in TCGA dataset (Fig. 3G). And expression analysis by qRT-PCR as well as by western blot analysis demonstrated CDK2 expression was markedly reduced in NR1H4 knockdown cells (Fig. 3H, I). Therefore, we supposed si-NR1H4 suppressed malignant phenotype of ccRCC cells due to the downregulation of CCNE2/CDK2 which could help cancer cells switch from G0/G1 to S phase.

**Table 3:** Enrichment of KEGG pathways in the NR1H4 high expression group

No		GS DETAILS	SIZE	ES	NES	NOM p-val	FDR q-val
1	KEGG_N_GLYCAN_BIOSYNTHESIS		42	0.602	2.223	0.000	0.000
2	KEGG_PROTEASOME		38	0.578	1.949	0.000	0.000
3	KEGG_SNARE_INTERACTIONS_IN_VESICULAR_TRANSPORT		33	0.574	1.702	0.250	0.013
4	KEGG_CELL_CYCLE		100	0.537	1.763	0.000	0.016
5	KEGG_MISMATCH_REPAIR		19	0.625	1.848	0.000	0.020
6	KEGG_DNA_REPLICATION		32	0.640	1.876	0.000	0.027
7	KEGG_P53_SIGNALING_PATHWAY		59	0.622	1.581	0.000	0.095
8	KEGG_NUCLEOTIDE_EXCISION_REPAIR		37	0.569	1.582	0.000	0.107
9	KEGG_PROTEIN_EXPORT		19	0.614	1.552	0.000	0.116
10	KEGG_RENAL_CELL_CARCINOMA		59	0.513	1.584	0.000	0.122
11	KEGG_HOMOLOGOUS_RECOMBINATION		20	0.402	1.554	0.000	0.128
12	KEGG_BASAL_TRANSCRIPTION_FACTORS		26	0.539	1.509	0.000	0.141
13	KEGG_TYPE_II_DIABETES_MELLITUS		43	0.334	1.487	0.000	0.141
14	KEGG_CHRONIC_MYELOID_LEUKEMIA		62	0.463	1.485	0.000	0.143
15	KEGG_BLADDER_CANCER		37	0.573	1.492	0.000	0.150
16	KEGG_MTOR_SIGNALING_PATHWAY		46	0.424	1.488	0.000	0.150
17	KEGG_BIOSYNTHESIS_OF_UNSATURATED_FATTY_ACIDS		17	0.634	1.522	0.000	0.152
18	KEGG_PANCREATIC_CANCER		64	0.509	1.523	0.000	0.164
19	KEGG_APOPTOSIS		81	0.470	1.451	0.200	0.230
20	KEGG_RIG_I_LIKE_RECEPTOR_SIGNALING_PATHWAY		58	0.374	1.433	0.000	0.268

Statistical data were performed by GSEA software

Abbreviations: *KEGG* Kyoto encyclopedia of genes and genomes, *ES* Enrichment score, *FDR q‐val* False discovery rate q value, *NES* Normal enrichment score, *NOM p‐val* Nominal P‐value

### Diagnostic and prognostic value of NR1H4 in ccRCC

**Fig. 4:**
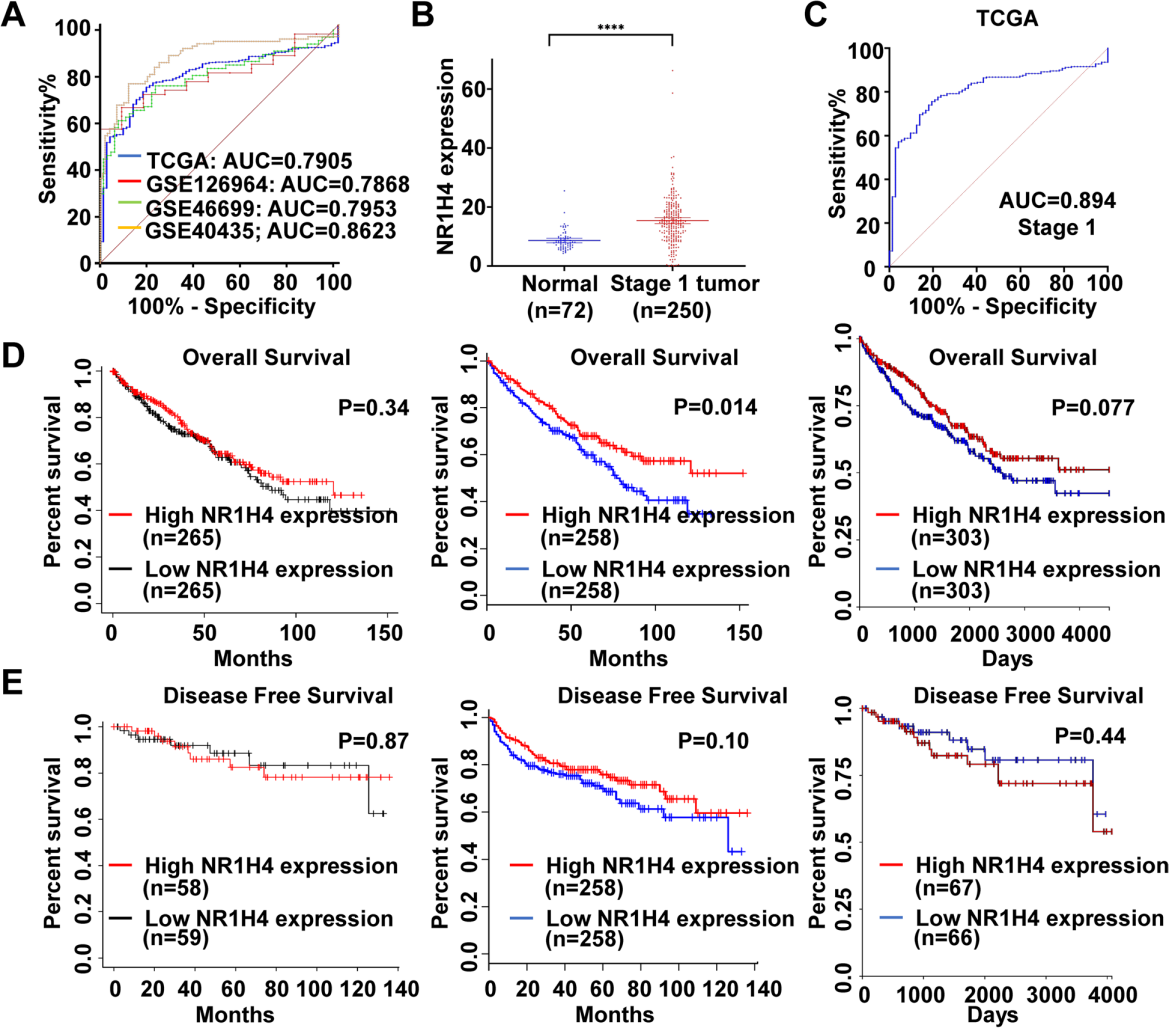
Diagnostic and prognostic value of NR1H4 in ccRCC. **A** ROC curves for ccRCC patients in TCGA and GEO datasets. **B** NR1H4 expression in normal tissues and stage I ccRCC in TCGA datasets. **C** ROC curve for stage I ccRCC patients in TCGA datasets. **D** OS survival curves of ccRCC patients in Kaplan‐Meier Plotter database, GEPIA database and UCSC Xena database. **E** DFS survival curves of ccRCC patients in Kaplan‐Meier Plotter database, GEPIA database and UCSC Xena database. ROC curve, receiver operating characteristic curve; AUC, area under the receiver operating characteristic curve; OS, overall survival; DFS, disease free survival; HR, hazard ratio; CI, confidence interval; GEPIA, Gene Expression Profiling Interactive Analysis; ****p < 0.0001

Despite the diagnosis and treatment of ccRCC have changed remarkably rapidly, a notable proportion of patients are still diagnosed at locally advanced disease and distant metastases stage [34]. The TCGA and GEO datasets were used to explore the diagnostic potential of NR1H4 in ccRCC. Significant diagnostic accuracy was shown in TCGA-KIRC with AUC = 0.7905 (95% CI 0.7474–0.8336; P < 0.0001), GSE126964 with AUC = 0.7868 (95%CI 0.6762–0.8973; *P* = 0.0028), GSE46699 with AUC = 0.7953 (95%CI 0.7158–0.8748; *P* < 0.0001) and GSE40435 with AUC = 0.8623 (95%CI 0.8081–0.9165; *P* < 0.0001) (Fig. 4A). Furthermore, NR1H4 was significantly overexpressed in stage I ccRCC patients (Fig. 4B). We separated the stage I patients to analyzed the diagnostic value of NR1H4. As shown in Fig. 4C, NR1H4 also showed a high diagnostic accuracy with AUC = 0.8094 (95%CI 0.7596–0.8593; *P* < 0.0001). These results revealed that NR1H4 possesses a high diagnostic potential in differentiating ccRCC patients from normal individuals, even for the early stages of ccRCC. To investigate the prognostic value of NR1H4, the Kaplan–Meier Plotter database, GEPIA database and UCSC Xena database were used. However, we found patients with higher expression of NR1H4 did not have poorer OS (overall survival) or DFS (disease free survival) (Fig. 4D, E).

### Genetic mutation and promoter methylation of NR1H4

**Fig. 5:**
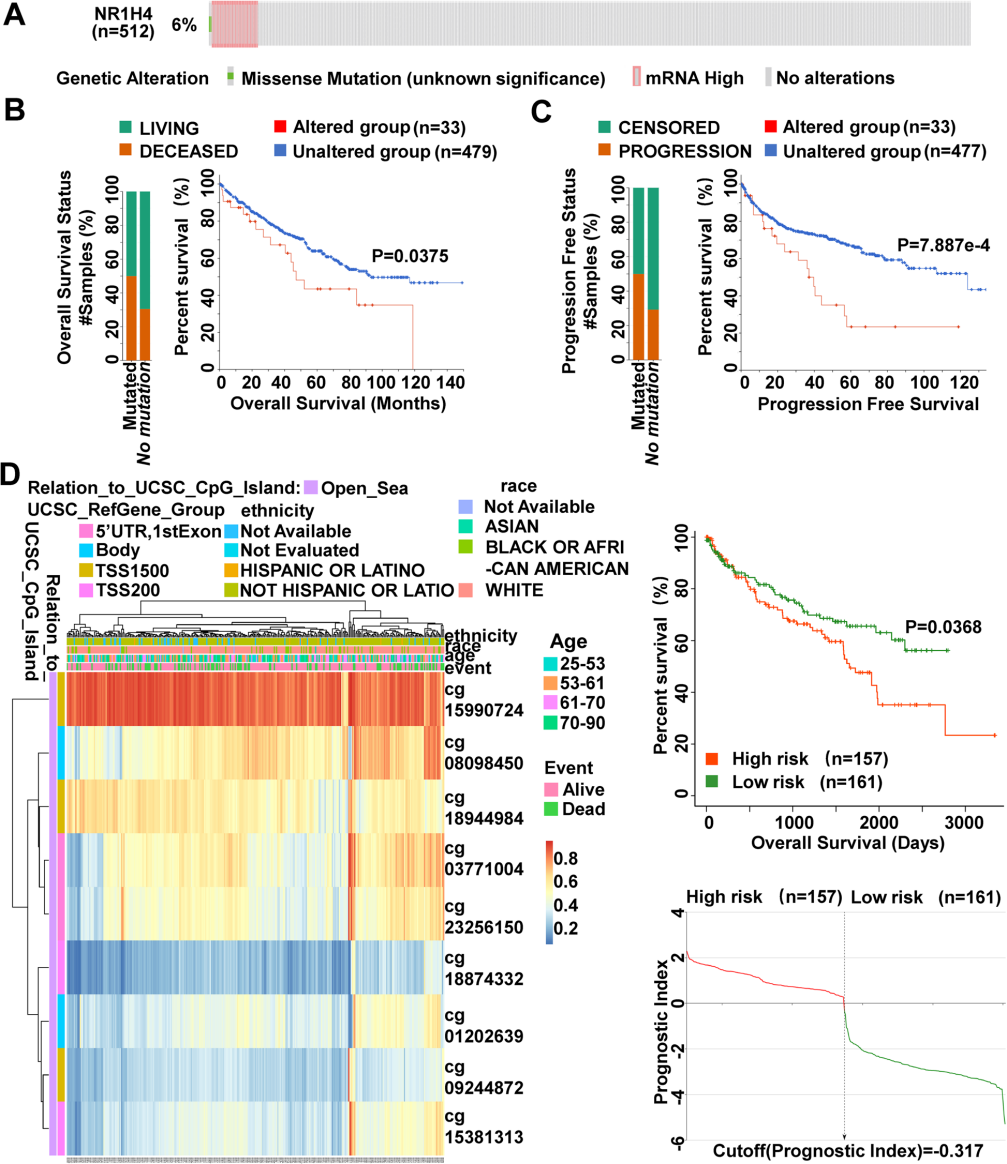
Genomic alterations and promoter methylation of NR1H4. **A** 6% mutation rate of NR1H4 was observed in ccRCC patients. Genetic alterations in NR1H4 were associated with shorter OS (**B**) and PFS (**C**) of ccRCC patients. **D** The DNA methylation level of NR1H4 in ccRCC patients and the Kaplan–Meier curves for survival analysis of DNA methylation of NR1H4 between high- and low-risk groups

**Fig. 6:**
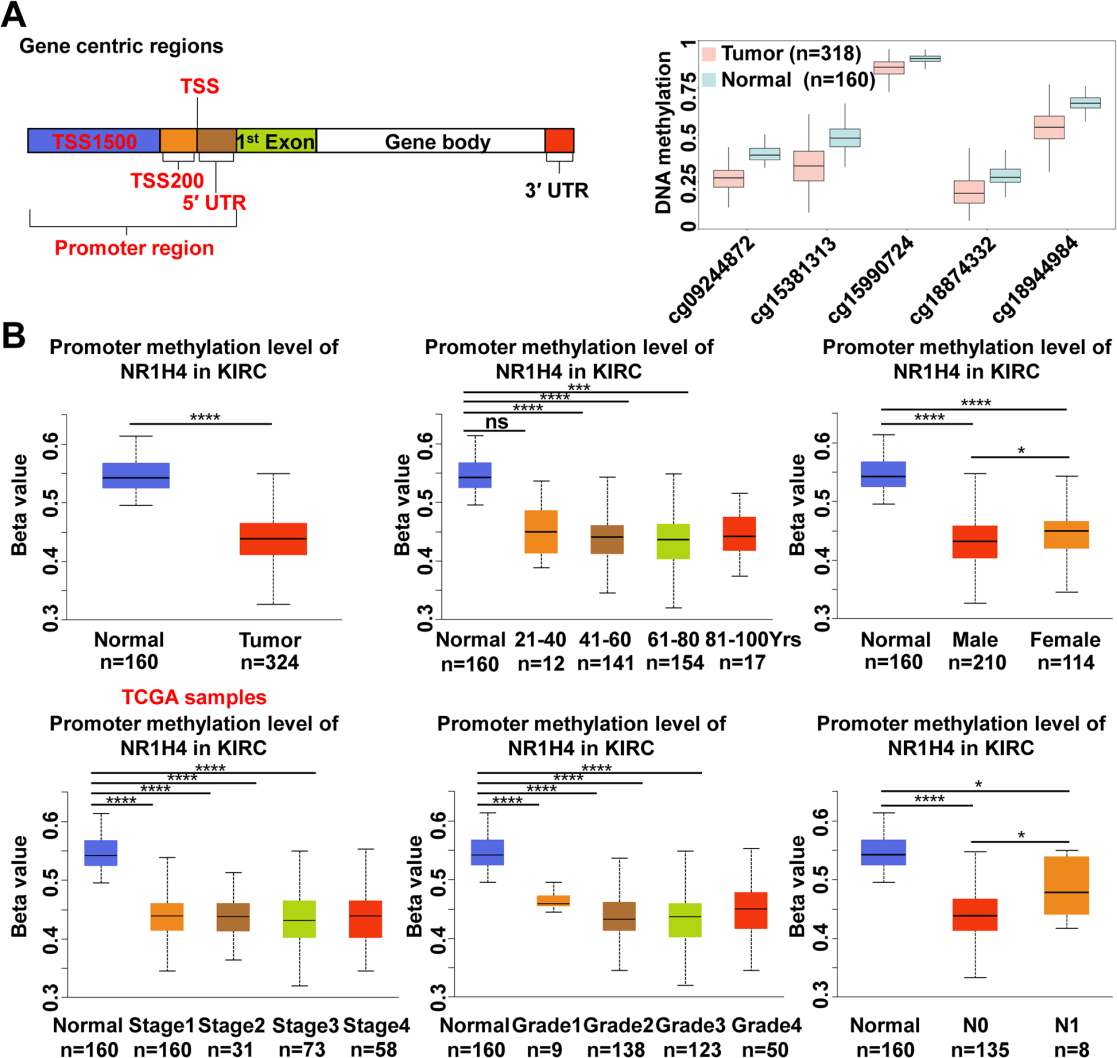
Promoter methylation of NR1H4. **A** The DNA methylation level of CpG sites in the promoter region. **B** Promoter methylation levels of NR1H4 in ccRCC patients based on different variables including sample types, patient’s age, patient’s gender, individual cancer stages, tumor grade and nodal metastasis status which determined using UALCAN. *p < 0.05; **p < 0.01; ***p < 0.001; ****p < 0.0001

Specific genetic mutations and epigenetically disrupted genes are good candidate targets for prognostic and diagnostic tools and treatment strategy [35, 36]. The cBioPortal database was used to analyzed genetic alteration in NR1H4 and its associations with OS and PFS (progression free survival) of ccRCC patients. In the 512 patients, genetic alteration was found in 33 ccRCC patients and the mutation rate was 6% (Fig. 5A). The results from Kaplan–Meier plot and log-rank test indicated that the higher genetic alteration in NR1H4 was associated with shorter OS (*P* = 0.0375) and PFS (*P* = 7.887e-4) of ccRCC patients (Fig. 5B, C). The heatmap of DNA methylation of NR1H4 was explored in MethSurv. Cluster analysis of single CpG islands in NR1H4 gene was performed in the form of heatmap, where methylation levels were combined with available patient characteristics and gene subregions. Among them, cg15990724 showed the highest DNA methylation level (Fig. 5D). To further explore the effect of NR1H4 DNA methylation on ccRCC patients’ prognosis, we divided ccRCC patients into two groups according to the risk of NR1H4 DNA methylation, and the survival analysis demonstrated that ccRCC patients in the low-risk group had longer survival times (p < 0.05) in SurvivalMeth (Fig. 5D). As shown in Fig. 6A, the CpG islands located in the NR1H4 promoter region showed lower DNA methylation level in ccRCC patients. Using UALCAN, we also found that promoter methylation of NR1H4 in sample types, patient age, patient gender, individual cancer stages, tumor grade and nodal metastasis status was reduced than normal tissues (Fig. 6B). DNA demethylation may be a contributing factor to NR1H4 overexpression. These results implied that genetic mutations and methylations of NR1H4 may significantly affect the prognosis of ccRCC patients.

### NR1H4 expression is associated with tumor immune infiltrates (TILs)

**Fig. 7:**
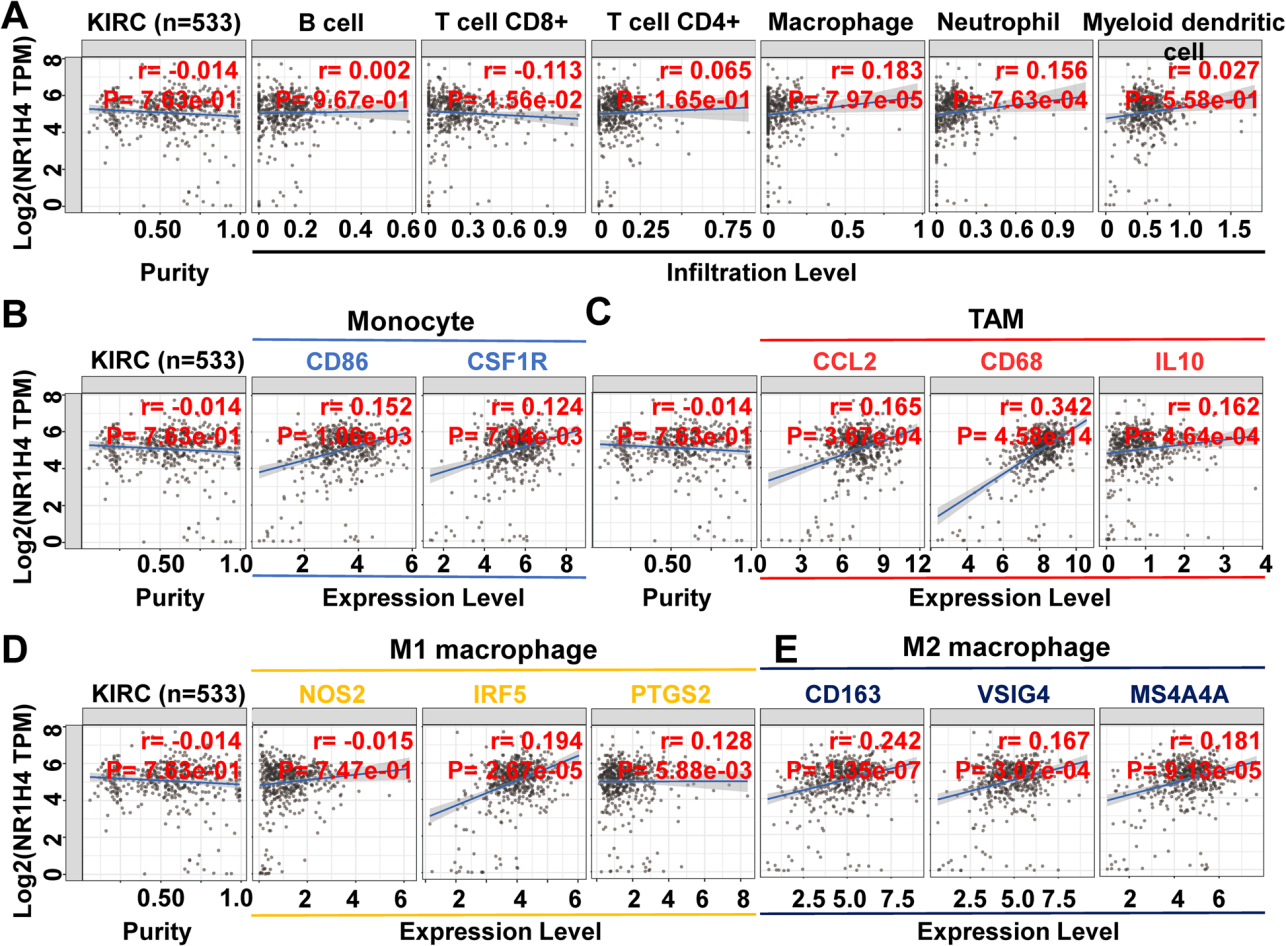
Correlation of NR1H4 expression with immune infiltration level in ccRCC. **A** NR1H4 is related to tumor purity and immune infiltration levels of ccRCC by TIMER analysis. Scatterplots of correlations between NR1H4 expression and gene markers of monocytes (**B**), TAM (**C**), and M1 (**D**) and M2 macrophages (**E**) in ccRCC. TAM, tumor-associated macrophage

Immunotherapy plays a significant role in kidney cancer treatment[37]. TIMER algorithm was performed in TIMER2.0 database to investigate the relationship between NR1H4 expression and tumor-infiltrating lymphocytes in ccRCC. The results showed that NR1H4 expression was positively associated with macrophage (r = 0.183, *p* = 7.97e-05) and neutrophil (r = 0.156, *p* = 7.63e-04) infiltration levels (Fig. 6A). CD8 + T cell infiltration levels were negatively correlated with NR1H4 expression (r = -0.113, *p* = 1.56e-02) (Fig. 7A). To further confirm the relationship between NR1H4 expression and immune cell infiltration levels in ccRCC, we used Spearman correlation analysis to explore the correlations between NR1H4 and immune markers of immune cells in TIMER2.0, and the purity-adjusted partial spearman's rho value as the degree of their correlation. The results showed there was a significant correlation between NR1H4 expression and the most of marker set of monocyte, Tumor-associated macrophages (TAM), M2 macrophage, Th2, Tfh, Treg and T cell exhaustion (Table 4). Specifically, we found that expression of NR1H4 was significantly correlated with chemokine (C–C motif) ligand (CCL)-2, CD68, IL10 of TAMs, especially CD163, VSIG4 and MS4A4A of M2 Macrophage in ccRCC (*P* < 0.001) (Fig. 7B-E). TISIDB and XCELL algorithm were used to further confirm the relationship between NR1H4 expression and immune cell infiltration levels in ccRCC. The results analyzed by TISIDB showed that NR1H4 expression was positively associated with active CD4 + T cell (r = 0.149, *p* = 0.000563), neutrophil (r = 0.143, *p* = 0.000928) and active dendritic cell (r = 0.125, *p* = 0.00386) infiltration levels (Additional file 3: Fig. S1A). Active B cell infiltration levels were negatively correlated with NR1H4 expression (r = -0.092, *p* = 0.0336) (Additional file 3: Fig. S1A). However, due to the different algorithm, there was no significant correlation between NR1H4 expression and macrophage cell infiltration levels in TISIDB (Additional file 3: Fig. S1A). Hence a third algorithm, the XCELL algorithm, was adopted. XCELL, a novel gene signature-based method, launched in 2017 by Aran’s group, to infer 64 immune and stromal cell types [38]. XCELL integrates the advantages of gene set enrichment with deconvolution approaches and could portray a full tumor microenvironment landscape across thousands of TCGA samples. The results analyzed by XCELL algorithm indicated that macrophage and M1 macrophage infiltration levels were not significantly correlated with NR1H4 expression, nevertheless, M2 macrophage infiltration levels were positively associated with NR1H4 expression (r = 0.118, *p* = 1.16e-02) (Additional file 3: Fig. S1B). These findings suggest that NR1H4 may regulate macrophage polarization and participate in the process of M2 type TAM infiltration in ccRCC. Thus, it may explain why NR1H4 predicts poor survival in ccRCC.

**Table 4:** Correlation analysis between NR1H4 and markers of immune cells in ccRCC by TIMER

Description	Gene markers	ccRCC				
		None			Purity	
		Correlation	p value		Correlation	p value
CD8 + T cell	CD8A	0.026	0.554		-0.030	0.514
	CD8B	-0.023	0.594		-0.091	0.051
T cell (general)	CD3D	-0.055	0.208		-0.112	0.016
	CD3E	-0.035	0.425		-0.088	0.060
	CD2	0.021	0.632		-0.031	0.513
B cell	CD19	-0.053	0.220		-0.082	0.077
	CD79A	-0.061	0.163		-0.116	0.013
Monocyte	CD86	0.172	***		0.152	*
	CD115 (CSF1R)	0.145	**		0.124	*
TAM	CCL2	0.162	**		0.165	**
	CD68	0.385	***		0.342	***
	IL10	0.169	***		0.162	**
M1 macrophage	INOS (NOS2)	0.022	0.616		-0.015	0.747
	IRF5	0.230	***		0.194	***
	COX2(PTGS2)	0.092	0.034		0.128	*
M2 macrophage	CD163	0.250	***		0.242	***
	VSIG4	0.189	***		0.167	**
	MS4A4A	0.186	***		0.181	***
Neutrophils	CD66b (CEACAM8)	0.040	0.362		0.041	0.379
	CD11b (ITGAM)	0.189	***		0.164	**
	CCR7	0.050	0.249		0.022	0.637
Natural killer cell	KIR2DL1	0.027	0.530		0.024	0.605
	KIR2DL3	-0.009	0.840		-0.024	0.602
	KIR2DL4	0.003	0.948		-0.002	0.967
	KIR3DL1	0.002	0.971		-0.017	0.719
	KIR3DL2	-0.030	0.494		-0.046	0.328
	KIR3DL3	0.002	0.970		0.008	0.858
	KIR2DS4	-0.004	0.921		-0.006	0.890
Dendritic cell	HLA-DPB1	0.092	0.034		0.039	0.408
	HLA-DQB1	0.064	0.140		-0.005	0.915
	HLA-DRA	0.179	***		0.137	*
	HLA-DPA1	0.177	***		0.146	*
	BDCA-1 (CD1C)	0.068	0.116		0.039	0.398
	BDCA-4 (NRP1)	0.302	***		0.296	***
	CD11c (ITGAX)	0.045	0.300		0.042	0.367
Th1	T-bet (TBX21)	-0.070	0.107		-0.103	0.027
	STAT4	0.102	0.019		0.077	0.100
	STAT1	0.257	***		0.227	***
	IFN-γ (IFNG)	0.008	0.849		-0.046	0.329
	TNF-α (TNF)	-0.008	0.849		-0.019	0.679
Th2	GATA3	-0.302	***		-0.318	***
	STAT6	0.380	***		0.370	***
	STAT5A	0.085	0.051		0.065	0.165
	IL13	0.033	0.454		0.023	0.628
T fh	BCL6	0.213	***		0.206	***
	IL21	-0.002	0.967		-0.005	0.918
Th17	STAT3	0.316	***		0.302	***
	IL17A	0.040	0.356		0.067	0.152
Treg	FOXP3	0.028	0.518		0.011	0.814
	CCR8	0.178	***		0.158	**
	STAT5B	0.276	***		0.256	***
	TGF-β (TGFB1)	0.083	0.054		0.069	0.140
T cell exhaustion	PD-1 (PDCD1)	-0.026	0.548		-0.079	0.091
	CTLA4	0.055	0.205		0.020	0.671
	TIM-3 (HAVCR2)	0.210	***		0.167	**
	GZMB	-0.178	***		-0.227	***
	LAG3	-0.046	0.291		-0.093	0.046
	PDL1 (CD274)	0.233	***		0.245	***

*TAM* Tumor-associated macrophage, *Th* T helper cell, *Tfh* Follicular helper T cell, *Treg* Regulatory T cell, None, correlation without adjustment. Purity, correlation adjusted by purity

^*^ p < 0.01

^**^ p < 0.001

^***^ p < 0.0001

## Discussion

NR1H4, a member belonging to the superfamily of nuclear receptors, encodes a ligand-activated transcription factor. Recently, the research on the role of NR1H4 in cancer has been investigated in several tumors. So far, NR1H4 is considered to act as a molecular mediator regulating tumorigenesis [39]. However, its expression pattern, clinical value and biological function in ccRCC remains obscure. Our result provides insights in understanding the pathologic role of NR1H4 in promoting tumor progression, as well as its potential value as a new diagnostic biomarker and therapeutic target for ccRCC.

In the present study, database analysis and patient sample detection revealed an increase expression of NR1H4 in ccRCC, especially in ccRCC tissue of stage I vs normal renal tissue. According to the ROC curve results, NR1H4 possesses high diagnostic value in distinguishing ccRCC patients from healthy individuals, which indicated that NR1H4 could serve as a potential biomarker for diagnosis. In addition, Kaplan–Meier test showed that high expression of NR1H4 mRNA was not significantly associated with poor OS or DFS in patients with ccRCC (Fig. 4; Additional file 2: Table S2). However, genetic alterations in NR1H4 were associated with poorer OS and PFS. Further investigation is needed to verify the prognosis of NR1H4. Next, we had investigated DNA methylation status through the MethSurv, SurvivalMeth and UALCAN databases, where promoter methylation of NR1H4 in ccRCC was attenuated than normal tissues based on sample types, patient’s age, patient’s gender, individual cancer stages, tumor grade and nodal metastasis status. The multi-omics strategy reveals a clinic significance of NR1H4 in ccRCC.

Studies on the function of NR1H4 in tumor progression are increased in recent years. For example, NR1H4 induces cell death and sensitizes to TRAIL-induced inhibition of growth in colorectal cancer cells through the up-regulation of death receptor 5 [40]. NR1H4 antagonizes Wnt/β-catenin signaling in colorectal tumorigenesis [31]. By promoting the binding of HDAC3 to NR1H4 promoter, the nuclear translocation of transketolase inhibits the farnesoid receptor expression in HCC [41]. NR1H4 upregulates the microRNA-23b-3p to regulate the proliferation and apoptosis of osteosarcoma cells [42]. To further evaluate the function of NR1H4 in ccRCC, we performed data analysis using GSEA software and Flow Cytometry. The results of CCK‐8, clone formation, EdU, transwell and wound healing assays indicated knockdown of NR1H4 could suppress the proliferation, migration and invasion of ccRCC cells. The cell cycle machinery orchestrates cell division. The key components of this machinery are cyclins and their associated catalytic partners, the cyclin-dependent kinases (CDKs) [43]. Correlation analysis and cell biological experiments showed that NR1H4 expression was positively associated with Cyclin E2 and CDK2, which indicated NR1H4 may play its oncogenic role in cancer by regulating CCNE2. However, due to the limitations of time and conditions, we did not further investigate how the CCNE2 and CDK2 were downregulated in NRIH4 knockdown cells at mRNA levels. Nevertheless, we are confident that this study could provide new ideas for more researchers to explore how NR1H4 regulates CCNE2 and CDK2 from the perspectives of transcriptional level, post-transcriptional level and epigenetic level. The detailed mechanism needs to be further elucidated.

Recently, tumor-associated immune cells and tumor immunotherapy have attracted much attention [44, 45]. The role of the immune system in cancer development and progression has been increasing recognized. Some studies have shown that immune cell infiltration has an influence on survival in ccRCC [46, 47]. Our results revealed a most significantly positive correlation between NR1H4 expression and the level of CD4 + T cell, macrophage/Monocyte and neutrophil infiltration levels in ccRCC. Moreover, the correlation between NR1H4 expression and the marker genes of immune cells implicate the role of NR1H4 in regulating tumor immunology in ccRCC. TAMs play key roles in tumor metastasis and therapeutic resistance, which often promote the progression of untreated tumors [48, 49]. TAMs are significantly plastic that can be either tumor-supportive (M2 macrophages) or tumoricidal (M1 macrophages) [50]. We suppose that NR1H4 may participate in the process of M2 type TAM infiltration. Inhibition of NR1H4 may reduce the infiltration of TAM, especially the M2 TAM infiltration, which may become a new idea for immunotherapy.

## Conclusions

In conclusion, this study indicates NR1H4 is a promising potential diagnostic and immune-related therapeutic target for ccRCC. Besides, genetic alteration and DNA methylation status demonstrated that NR1H4 has significant prognostic and clinicopathological value in ccRCC. Knockdown of NR1H4 could suppress progression of ccRCC and induce downregulation of CCNE2. Further studies are needed to confirm these results and reveal the underlying mechanisms.

## Supplementary Information

**Additional file 1:** **Table S1**. Indicated primers used in PCR experiments.

**Additional file 2:** **Table S2**. Univariate and multivariate analyses for Clear Cell Renal Cell Cancer patients on overall survival in the TCGA.

**Additional file 3:** **Figure S1**. The relationship between NR1H4 expression and immune cell infiltration levels in ccRCC. (A) The correlations between NR1H4 expression and immune infiltration levels of ccRCC by TISIDB database analysis. (B) The correlations between NR1H4 expression and macrophage infiltration levels by XCELL algorithm analysis.

**Additional file 4**. Original blots in the manuscript.

## Data Availability

The datasets analyzed during the current study are available in the The Cancer Genome Atlas (TCGA) (https://portal.gdc.cancer.gov), Gene Expression Omnibus (GEO) database (https://www.ncbi.nlm.nih.gov/geo), Tumor Immune Estimation Resource 2.0 (http://timer.cistrome.org/), GEPIA (http://gepia.cancer-pku.cn/index.html), UALCAN (http://ualcan.path.uab.edu/index.html), The Kaplan‐Meier Plotter (http://www.kmplot.com), UCSC Xena (https://xena.ucsc.edu/), The cBio Cancer Genomics Portal (http://cbioportal.org), MethSurv (https://biit.cs.ut.ee/methsurv/), and SurvivalMeth (http://bio-bigdata.hrbmu.edu.cn/survivalmeth/). All data generated or analyzed during this study are included in this published article (and its supplementary information files). All the data were available from the corresponding authors for reasonable request.

## Abbreviations

NR1H4: Nuclear receptor subfamily 1 group H member 4
FXR: Farnesoid X receptor
ccRCC: Clear cell renal cell carcinoma
TIMER: Tumor immune estimation resource
CRC: Colorectal cancer
HCC: Hepatocellular carcinoma
NSCLC: Non-small cell lung cancer
ACC: Adrenocortical carcinoma
BLCA: Bladder urothelial carcinoma
BRCA: Breast invasive carcinoma
CESC: Cervical squamous cell carcinoma and endocervical adenocarcinoma
CHOL: Cholangiocarcinoma
COAD: Colon adenocarcinoma
DLBC: Lymphoid neoplasm diffuse large B cell lymphoma
ESCA: Esophageal carcinoma
GBM: Glioblastoma multiforme
HNSC: Head and neck squamous cell carcinoma
KICH: Kidney chromophobe
KIRC: Kidney renal clear cell carcinoma
KIRP: Kidney renal papillary cell carcinoma
LAML: Acute myeloid leukemia
LGG: Lower grade glioma
LIHC: Liver hepatocellular carcinoma
LUAD: Lung adenocarcinoma
LUSC: Lung squamous cell carcinoma
MESO: Mesothelioma
OV: Ovarian serous cystadenocarcinoma
PAAD: Pancreatic adenocarcinoma
PCPG: Pheochromocytoma and paraganglioma
PRAD: Prostate adenocarcinoma
READ: Rectum adenocarcinoma
SARC: Sarcoma
SKCM: Skin cutaneous melanoma
STAD: Stomach adenocarcinoma
TGCT: Testicular germ cell tumors
THCA: Thyroid carcinoma
THYM: Thymoma
UCEC: Uterine corpus endometrial carcinoma
UCS: Uterine carcinosarcoma
UVM: Uveal melanoma
GEO: Gene expression omnibus
TCGA: The cancer genome atlas
GAPDH: Glyceraldehyde-3-phosphate dehydrogenase
OD: Optical density
EdU: (5-Ethynyl-2' -deoxyuridine)
siRNA: Small interfering RNA
CCNA2: Cyclin A2
CCNB1: Cyclin B1
CCNB2: Cyclin B2
CCNB3: Cyclin B3
CCND1: Cyclin D1
CCND2: Cyclin D2
CCND3: Cyclin D3
CCNE1: Cyclin E1
CCNE2: Cyclin E2
CDK2: Cyclin dependent kinase 2
DAPI: 2-(4-Amidinophenyl)-6-indolecarbamidine dihydrochloride
KEGG: Kyoto encyclopedia of genes and genomes
ES: Enrichment score
FDR q‐val: False discovery rate q value
NES: Normal enrichment score
NOM p‐val: Nominal P‐value
ROC curve: Receiver operating characteristic curve
AUC: Area under the receiver operating characteristic curve
OS: Overall survival
DFS: Disease free survival
PFS: Progression free survival
HR: Hazard ratio
CI: Confidence interval
GEPIA: Gene expression profiling interactive analysis
TAM: Tumor-associated macrophage
Th: T helper cell
Tfh: Follicular helper T cell
Treg: Regulatory T cell
FBS: Fetal bovine serum
cDNA: DNA complementary to RNA
PAGE: PA-gel electrophoresis
SDS: Sodium dodecyl sulfate
PVDF: Polyvinylidene fluoride
PBS: Phosphate buffer saline
CCK-8: Cell counting kit-8
GSEA: Gene set enrichment analysis
IHC: Immunohistochemistry
qRT-PCR: Quantitative real‐time polymerase chain reaction
SD: Standard deviation
ANOVA: Analysis of variance
