# Supplementary material for: Clinical significance and oncogenic function of NR1H4 in clear cell renal cell carcinoma
Source: BMC Cancer. 2022 Sep 19;22:995. doi: 10.1186/s12885-022-10087-4 (PMC9487048; doi:10.1186/s12885-022-10087-4)
Supplement: Supplementary file 1 — Additional file 1: Table S1. Indicated primers used in PCR experiments. [file 12885_2022_10087_MOESM1_ESM.pdf]

Additional file 1: Table S1 Indicated primers used in PCR experiments.

GENE	Primer sequences (5′-3′)
NR1H4	F: GATTGCTTTGCTGAAAGGGTC
	R: CAGAATGCCCAGACGGAAG
GAPDH	F: ATCATCCCTGCCTCTACTGG
	R: GTCAGGTCCACCACTGACAC
CCNB3	F: ATGAAGGCAGTATGCAAGAAGG
	R: CATCCACACGAGGTGAGTTGT
CCND1	F: ATGGAACACCAGCTCCTGTG
	R: ACCTCCAGCATCCAGGTGGC
CCND2	F: TCCAAACTCAAAGAGACCAGC
	R: TTCCACTTCAACTTCCCCAG
CCNE2	F: GCATTATGACACCACCGAAGA
	R: TAGGGCAATCAATCAATCACAGC
CDK2	F: CCCTTTCTTCCAGGATGTGA
	R: TGAGTCCAAATAGCCCAAGG
