# Supplementary material for: Clinical significance and oncogenic function of NR1H4 in clear cell renal cell carcinoma
Source: BMC Cancer. 2022 Sep 19;22:995. doi: 10.1186/s12885-022-10087-4 (PMC9487048; doi:10.1186/s12885-022-10087-4)
Supplement: Supplementary file 2 — Additional file 2: Table S2. Univariate and multivariate analyses for Clear Cell Renal Cell Cancer patients on overall survival in the TCGA. [file 12885_2022_10087_MOESM2_ESM.pdf]

Additional file 2: Table S2 Univariate and multivariate analyses for Clear Cell Renal Cell Cancer patients on overall survival in the TCGA.

Variable	Univariate analysis		Multivariate analysis	
	HR (95% CI)	p-value	HR (95% CI)	p-value
Age				
≤ 55 vs >5 5 years	1.849(1.277-2.677)	0.001	1.463(1.004-2.132)	0.048
Gender				
male vs Female	1.059(0.762-1.471)	0.734		
AJCC stage				
Ⅰ vs Ⅱ vs Ⅲ vsⅣ	1.946(1.692-2.238)	0.000	1.664(1.418-1.954)	0.000
Grade				
1 vs 2 vs 3 vs 4	2.460(1.973-3.066)	0.000	1.610(1.263-2.051)	0.000
NR1H4 expression				
Low vs High	0.864(0.629-1.186)	0.366		
