# Supplementary material for: Clinical significance and oncogenic function of NR1H4 in clear cell renal cell carcinoma
Source: BMC Cancer. 2022 Sep 19;22:995. doi: 10.1186/s12885-022-10087-4 (PMC9487048; doi:10.1186/s12885-022-10087-4)
Supplement: Supplementary file 3 — Additional file 3: Figure S1. The relationship between NR1H4 expression and immune cell infiltration levels in ccRCC. (A) The correlations between NR1H4 expression and immune infiltration levels of ccRCC by TISIDB database analysis. (B) The correlations between NR1H4 expression and macrophage infiltration levels by XCELL algorithm analysis. [file 12885_2022_10087_MOESM3_ESM.pdf]

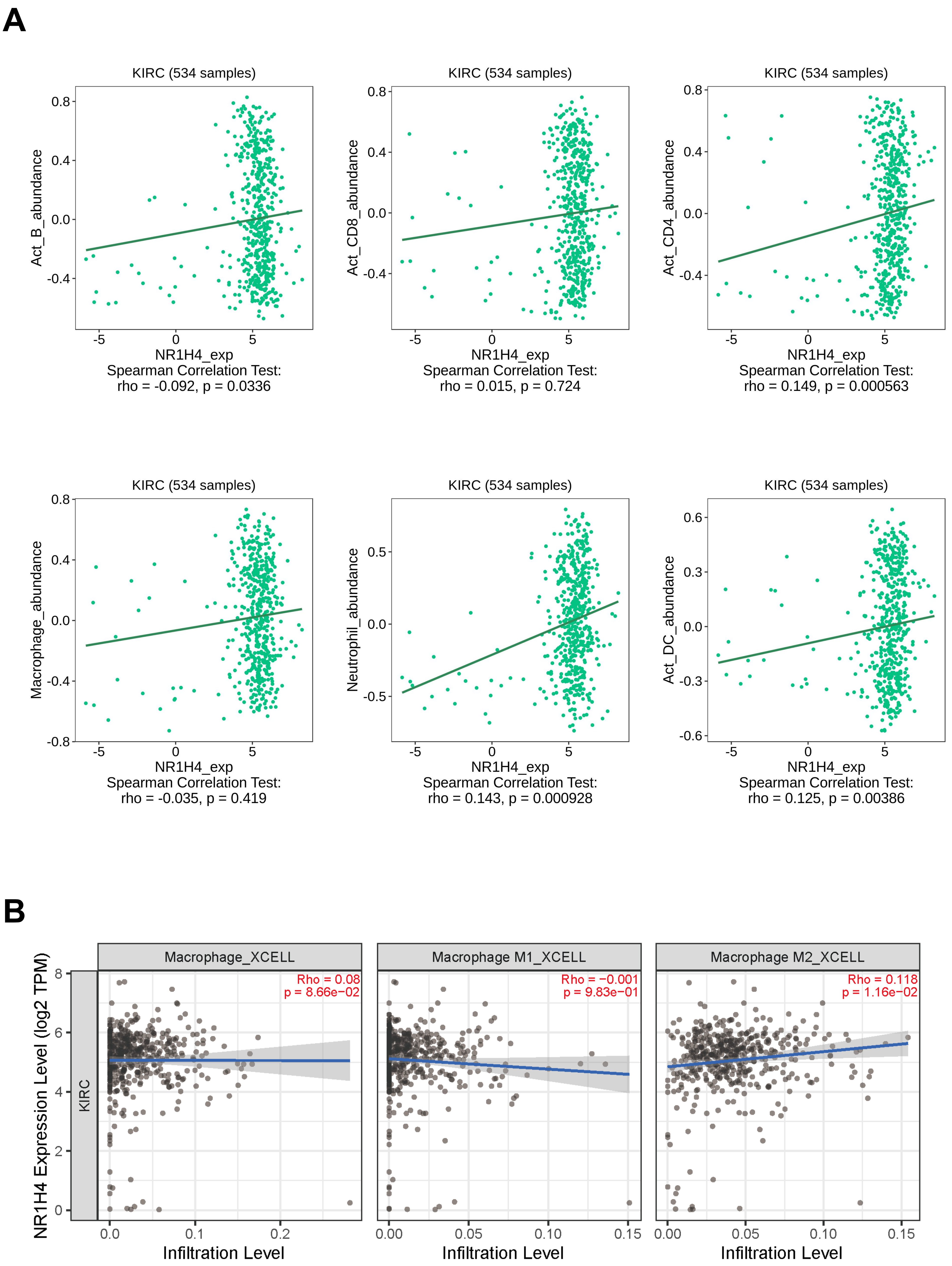

Fig. S1. The relationship between NR1H4 expression and immune cell infiltration levels in ccRCC. (A) The correlations between NR1H4 expression and immune infiltration levels of ccRCC by TISIDB database analysis. (B) The correlations between NR1H4 expression and macrophage infiltration levels by XCELL algorithm analysis.
