# Supplementary figures and images for: Clinical significance and oncogenic function of NR1H4 in clear cell renal cell carcinoma
Source: BMC Cancer. 2022 Sep 19;22:995. doi: 10.1186/s12885-022-10087-4 (PMC9487048; doi:10.1186/s12885-022-10087-4)

Original blots in the manuscript

Fig. 2D

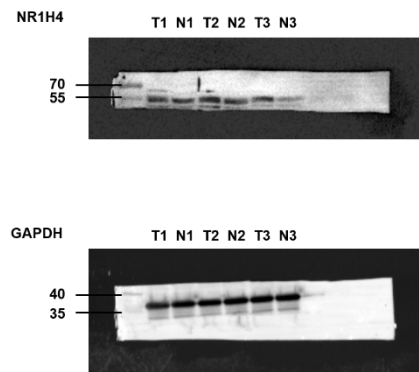

Fig. 2E

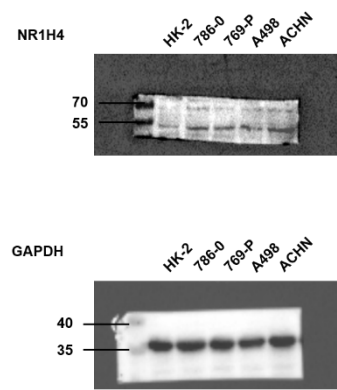

Fig. 2F

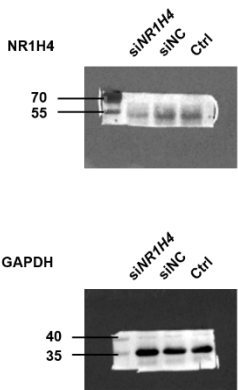

Fig. 3D

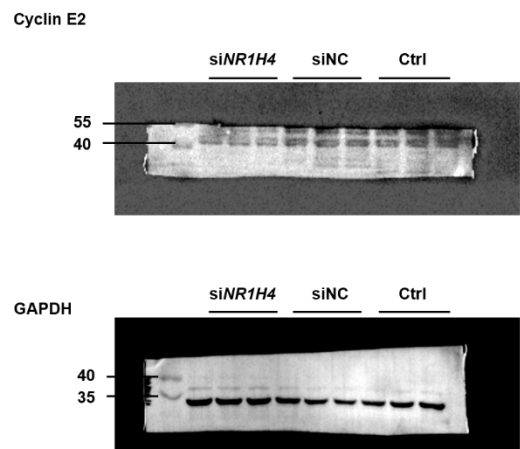

Fig. 3I

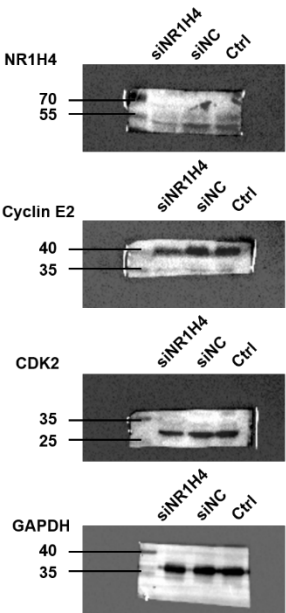

Supplement: Supplementary file 4 — Additional file 4. Original blots in the manuscript. [file 12885_2022_10087_MOESM4_ESM.pdf]
